# The History of Charing Cross Hospital and School

**Published:** 1914-07-25

**Authors:** 


### THE PORTRAIT OF A GREAT FOUNDER.

### The History of Charing Cross Hospital and School.

Dr. William Hunter, physician and director of pathology, and Dean of the Medical School, is an Edinburgh *alumnus* and graduate, but his enthusiasm and affection for his adopted hospital and school are fully as great as the most patriotic Charing Cross Hospital-man could desire. He has brought to his task of compiling the history of that hospital and its school* not only an unremitting industry, but also a practised pen and a fine sense of literary proportion. Fortunately his labours have been expended very largely upon a personality well worthy of immortality, the truly heroic figure of Benjamin Golding, founder of the hospital and school. He it was who first conceived in 1815, at the early age of twenty-one, the then quite unique proposition of a closely correlated hospital and school, which were brought into existence three years later and watched over by their founder for nearly fifty years.

The distinguishing feature of the plan which Dr. Golding evolved was a medical school upon the foundation where practical information may be conjoined with scientific instruction to those pupils and professional students who may be desirous of pursuing their education there, a proportion of the pecuniary receipts thence arising to be appropriated exclusively to the funds of the charity." In 1822 a "plan" of education, drawn up by Dr. Golding—still under thirty years of age—was adopted. It provided, amongst other things, for the giving of lectures upon every branch of medicine and its auxiliary sciences by the members of the professional staff deemed by their colleagues best qualified to give them. The extraordinary foresight of Golding is well shown by the list of subjects in which he desired instruction to be given, for it includes, besides the ordinary subjects of medical education a hundred years ago, tropical diseases, mental diseases, orthopædics, balneology, medical electricity, dietetics, naval and military surgery. Almost the only development of modern medicine which this wonderful man did not foresee was that of hygiene and preventive medicine, unless we add radium and vaccine therapy to it. Further, the "plan" actually proposed to supply the want of a University in London, so far as medical education was concerned. In the words of Dr. Hunter, this plan, "wide in its scope and replete with University ideals . . . is unique in its conception, its boldness, its breadth of view. . . . It includes every object, and even every subject, every principle of action and of honourable conduct, every motive of humanity, and every principle in the extension of medical knowledge that underlie, or have ever underlain, the great purposes and objects of medical education." By going direct to the statutes of Dr. Golding, and to original documents of all sorts in connection with the foundation of the hospital and school, Dr. Hunter is able to present an absolutely authentic record of the early history of both, and to draw a fine portrait of the great founder, than whom no man of his age did more to mould the institutional world as it exists to-day.

The original name of the hospital was the Royal West London Infirmary and Lying-in Institution, Charing Cross. Indeed, the provisional title first decided on was simply the West London Infirmary. It was in 1827—six years after the first general meeting of governors and twelve years after the initial and tentative opening—that the name was changed to that of the Charing Cross Hospital. During the 'thirties the young institution with its school passed through a time of very considerable stress, but under Golding's sagacious direction it emerged triumphant, and has never since looked back. In 1834 the hospital and school were accommodated in buildings specially designed for them on land leased from the Government at a heavy ground rent, and built at a cost of about £20,000. The freehold was purchased in 1850. About the same time (1833) Queen Victoria, then Princess, became patron of the institution. There were about 136 beds in the institution at this period. It was not until 1862, the year preceding his death, that Golding relinquished the control of the administration. Long before that he had had the satisfaction of seeing his life-work firmly established in public favour and professional esteem. In 1840 this remarkable man was seriously ill of an apoplexy, and, though he recovered, he was left permanently paralysed on the left side and a cripple. He thereupon took a house adjacent to his beloved hospital, and had a door opened directly through, to give him easier access. It is characteristic of the man, says Dr. Hunter, that no mention of this disability is to be found in any of his records, and that he did not allow it to interrupt his unceasing exertions on behalf of his hospital up to the time of his death, twenty-two years later. In 1862 Dr. Golding resigned his post as physician to the hospital, being then in his sixty-ninth year. There was, of course, no age-limit for the hospital staff in those days; indeed, Dr. Shearman, one of the original staff, and physician to the hospital for over forty years, did not retire from his active work until he was eighty-five years of age! The last words to the Council, defining its future policy, which tradition assigns to its founder, were: "Gentlemen, fund your legacies."

Curiously enough, the two Charing Cross Hospital students who have so far left the most indelible mark on their times and achieved the greatest renown, both abandoned the active practice of medicine in favour of biology and of exploration respectively. They were T. H. Huxley and David Livingstone, two giants of the Victorian epoch hard to be matched by any other medical school in the kingdom. There were, needless to say, many

* *Historical Account of Charing Cross Hospital and Medical School.* By William Hunter, M.D., F.R.C.P. (London: John Murray. Pp. 309. Price 21s.)

distinguished physicians and surgeons educated at Golding's foundation as well. Five Presidents of the English Royal Colleges have been on the medical staff, but Dr. Hunter—rightly, we believe—evidently regards Huxley as the greatest man whom the school has so far produced. A special chapter is devoted to the career of this great genius, and it is clearly shown how his education at Charing Cross Hospital influenced his mind and his future.

Dr. Hunter's history has clearly been to him a labour of love, and it forms a very worthy memorial to the hospital, to the school, to their founder, and incidentally to himself. It is sumptuously produced in Mr. Murray's safe hands, and is illustrated by numerous plates. There are few institutions the histories of which have been compiled at once so lovingly, so thoroughly, and so skilfully as has that of Charing Cross Hospital.

